# Attosecond time-resolved photoelectron holography

**DOI:** 10.1038/s41467-018-05185-6

**Published:** 2018-07-18

**Authors:** G. Porat, G. Alon, S. Rozen, O. Pedatzur, M. Krüger, D. Azoury, A. Natan, G. Orenstein, B. D. Bruner, M. J. J. Vrakking, N. Dudovich

**Affiliations:** 1 JILA, National Institute of Standards and Technology and University of Colorado-Boulder, Boulder, CO 80309-0440 USA; 2 Department of Physics of Complex Systems, Weizmann Institute of Science, Rehovot, 76100 Israel; 3 Stanford PULSE Institute, SLAC National Accelerator Laboratory, Menlo Park, CA 94025 USA; 4 Max-Born-Institut, Max Born Strasse 2A, Berlin, 12489 Germany

## Abstract

Ultrafast strong-field physics provides insight into quantum phenomena that evolve on an attosecond time scale, the most fundamental of which is quantum tunneling. The tunneling process initiates a range of strong field phenomena such as high harmonic generation (HHG), laser-induced electron diffraction, double ionization and photoelectron holography—all evolving during a fraction of the optical cycle. Here we apply attosecond photoelectron holography as a method to resolve the temporal properties of the tunneling process. Adding a weak second harmonic (SH) field to a strong fundamental laser field enables us to reconstruct the ionization times of photoelectrons that play a role in the formation of a photoelectron hologram with attosecond precision. We decouple the contributions of the two arms of the hologram and resolve the subtle differences in their ionization times, separated by only a few tens of attoseconds.

## Abstract (web summary)

Field induced tunneling is one of the fundamental processes of light-matter interaction. Here the authors reconstruct the  temporal properties of tunneling using two-color electron holography with attosecond time resolution using argon atoms.

## Introduction

**Fig. 1:**
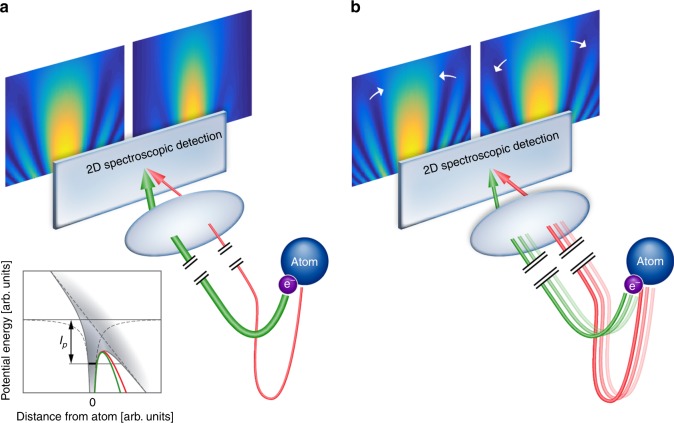
Perturbing the dynamics in photoelectron holography. The velocity map imaging (VMI) spectrometer maps direct (green) and rescattered (red) photoelectron trajectories with the same final momenta onto the same point of a position-sensitive detector. **a** The effect of the second harmonic on the amplitude of the electron trajectories causes the contrast of the interference pattern to vary with the two-color delay. Inset: Due to their different ionization times, the signal and reference electron trajectories experience different tunneling barriers, indicated by the green and red curves. **b** The effect of the SH on the phase gained along the electron trajectories in the continuum causes the positions of the interference peaks and dips to shift with the two-color delay

Laser-induced tunneling, one of the most basic strong field phenomena, serves as a starting point to attosecond science^1–11^. The coherent properties of the tunneled electron manifest themselves in time-resolved electron holography^12,31^. Here, holography is induced by the interference of two electron trajectories, both driven by the strong laser field. On the one hand, once the electron tunnel-ionizes, it is accelerated by the strong laser field, which dictates its final momentum. This electron trajectory serves as the hologram’s reference beam. Alternatively, the electron may scatter off the parent ion, serving as the signal beam. The holographic interference pattern of the two trajectories is observable in the photoelectron momentum distribution (see a schematic description in Fig. 1). Electron holography holds unique potential for probing the structural-temporal properties of an ionic system with Angstrom resolution and attosecond precision. To date, this approach has been applied to resolve structural information in aligned molecules^13–15^ and to identify different families of electron trajectories^16,17^; however, the detailed sub-cycle electron dynamics associated with the hologram have remained elusive.

The basic components of the hologram—the beam splitter, the two paths that represent its arms and the beam combiner—are all defined by the sub-cycle strong field dynamics. Within a semi-classical picture, they manifest in the complex ionization time and the action that the electron accumulates in the laser field^12,18,31^. Recording the hologram projects these properties onto the photoelectron momentum distribution, obscuring an intuitive insight into their attosecond dynamics. In this paper we demonstrate the ability to decouple the two arms of the interferometer that produces the hologram, and reconstruct their temporal properties. By adding a weak second harmonic (SH) field to the strong fundamental laser field, we induce changes in the hologram that depend on the relative phase of the fundamental and SH field, and infer the separate roles of the ionization time and the continuum electron dynamics^16,19–21^. This approach, previously applied to reveal the recollision dynamics that leads to the high harmonic generation process^20,21^, enables the reconstruction of the subcycle dynamics that underlies the hologram. Specifically, the SH field acts as a sub-cycle perturbation that probes the different interaction steps, decouples their contribution and reveals their underlying dynamics. Reconstructing the hologram’s dynamics is a first step towards the application of photoelectron holography as an advanced, attosecond-scale spectroscopic scheme.

## Results

### Two-color electron holography

We resolve the dynamical properties of the hologram by adding a weak perturbation in a parallel polarization configuration. Adding a weak perturbation modifies both the beam splitter, defining the relative strength of the signal and reference beams via control of the tunneling probability, and the length of each arm of the interferometer, via manipulation of the phase accumulated by the electron as it propagates in the continuum^20^. The subtle modifications of the tunneling probability translate into modifications of the related wavefunction amplitudes, leading to an enhancement or suppression of the contrast of the holographic interference (see Fig. 1a). Phase perturbations, associated with subtle modifications of the propagation in the continuum, are manifested in a slight displacement of the holographic fringe pattern with the two-color delay, modulating the regions of constructive or destructive interference (see Fig. 1b). Both the amplitude and phase perturbations are imprinted in the 3D holographic patterns that are formed during a two-color delay scan, and are measurable by their projection onto a 2D momentum detector.

In our experiment, we generate photoelectron holograms from argon atoms using a strong fundamental laser field with a wavelength of 788 nm and an intensity of 1.3 × 10^14^ W cm^−2^. The momentum distribution of the photoelectrons is measured using a velocity map imaging (VMI) spectrometer^22^. We perturb the hologram by adding the SH of the fundamental field, polarized parallel to the fundamental field. The intensity ratio of the SH to the fundamental is chosen to be less than 0.001, ensuring a weak perturbation. A detailed description of the experimental set up is given in Supplementary Note 8.

**Fig. 2:**
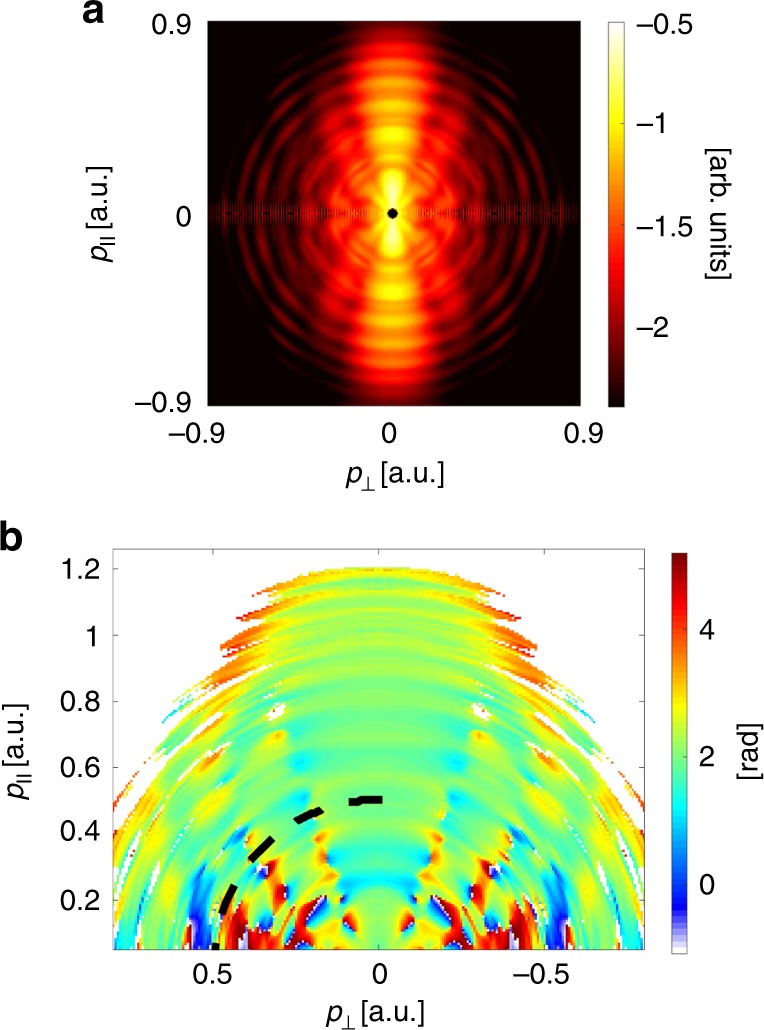
Two-color photoelectron holography. **a** Reconstructed photoelectron momentum distribution in log scale measured from argon, using the fundamental laser field only. The interference between the direct and scattered electron trajectories produces a spider-like interference pattern^12^. **b** Momentum-resolved two-color oscillation phase, *ϕ*_opt_(*p*_||_, *p*_⊥_), extracted from the two-color scan. The black dashed curve traces electrons with a constant absolute value of the momentum (**p** = 0.5 a.u.) and hence traces electrons with equal kinetic energy. The evolution of the two-color phase along this equal energy curve is used to extract the difference between the ionization times of the direct (reference) and scattered (signal) electron trajectories (see text)

Figure 2a displays an experimentally measured photoelectron hologram induced solely by the fundamental field, displayed as a function of the parallel and perpendicular momentum components, **p** = (*p*_||_, *p*_⊥_), defined with respect to the laser field’s polarization. The figure shows a 2D slice through the reconstructed 3D momentum distribution^23^. As has been previously shown, the measurement contains a spider-like interference pattern which stems from the interference between direct and scattered electron trajectories^12^. Introducing the weak SH field and varying the delay between the two colors leads to pronounced modulations of the holographic pattern. At each momentum value (*p*_||_, *p*_⊥_) the signal oscillates with the SH frequency. We analyze these modulations by extracting the value of the relative two-color phase, from hereon called *ϕ*_opt_(*p*_||_, *p*_⊥_), which maximizes the signal intensity (see Fig. 2b). Clearly, the oscillation phase changes across the hologram, and is distinctly different in neighboring regions where constructive and destructive interference occurs.

### Perturbative study of the direct electron trajectory

First, we focus on the main bright fringe, centered on *p*_⊥_ = 0. Along this fringe, the direct and scattered electron’s paths interfere constructively, encoding their average response. Since their ionization times are very similar (see Supplementary Note 3), one can assume the perturbation to have a similar effect on both (direct and scattered) amplitudes. Given that the direct electron's contribution is substantially larger than that of the scattered electron, we associate the oscillation phase, measured along *p*_⊥_ = 0, with the amplitude perturbation of the direct electron only (see Supplementary Note 5). A close examination of Fig. 2b shows that this phase slowly varies along *p*_||_. We investigate the origin of this variation and extract the dynamical information it contains.

The quantum mechanical description of the strong field laser ionization is dictated by the coherent addition of all quantum paths that contribute to the same observable, which in this case is the formation of a photoelectron with a particular final momentum. Along each path the electron acquires a phase that is given by the semi-classical action, *S*. In the strong field regime the action varies rapidly with time and therefore can be approximated by a stationary phase approximation (SPA)^18,24^. This approximation provides a direct mapping between each stationary ionization time *t*_0_ and a corresponding final momentum **p**. The real part of *t*_0_ is associated with the time at which the electron appears in the continuum, while the imaginary part is associated with the ionization amplitude^24^. Adding a weak SH field perturbs the semi-classical action according to $\tilde S(t_0,{\bf{p}},\phi )$ = *S*_0_(*t*_0_, **p**) + *δS*(*t*_0_, **p**, *ϕ*), where *S*_0_ is the unperturbed action in a fundamental-only laser field, *δS*(*t*_0_, **p**, *ϕ*) represents the first-order correction due to the SH field and *ϕ* is the two-color delay. The perturbation of the action maps the stationary ionization time *t*_0_ into an experimental observable, namely the modulation of the amplitude and phase of each trajectory with the two-color delay.

**Fig. 3:**
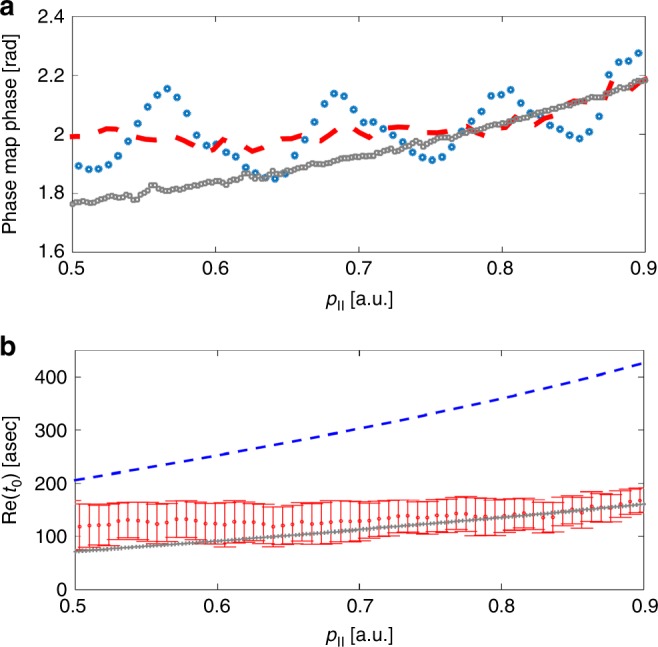
Reconstruction of the ionization time for direct electrons. **a** Experimentally measured two-color phase, extracted along *p*_⊥_ = 0 (blue circles) together with the CCSFA prediction (gray squares). The red dashed curve describes the experimental results after removing the oscillation associated with the ATI rings. **b** The reconstructed Re{*t*_0_} (red) and the CCSFA’s Re{*t*_0_} (gray) as a function of *p*_||_ for *p*_⊥_ = 0. The difference between the classical curve (blue dashed line) on the one hand, and the CCSFA curve and the experimental results on the other, reflects that the ionization times are confined to a substantially narrower range in the quantum mechanical picture. An estimation of the error bars is explained in Supplementary Note 5

Since along *p*_⊥_ = 0 the direct and scattered electrons interfere constructively, we record amplitude modulations only, dictated by Im{*δS*(*t*_0_, **p**, *ϕ*)}, representing the change in the ionization yield of—predominantly—the direct electrons. Figure 3a (blue circles) shows the experimentally measured *ϕ*_opt_ along *p*_⊥_ = 0, reflecting the two-color delay for which this yield is maximized. The periodic oscillation that is visible in *ϕ*_opt_ is related to above threshold ionization (ATI), induced by the interference between trajectories separated by one optical cycle^19,26^. Applying Fourier analysis we remove the periodic oscillations (dashed red line), isolating the single cycle response. In addition, we have evaluated this phase by a calculation (gray squares) using the Coulomb-corrected strong field approximation (CCSFA). This approximation is based on a semi-classical calculation of the momentum map performed by launching electron trajectories with initial conditions obtained by solution of the saddle-point equations governing the ionization process and by including the effect of the Coulomb interaction on the electron’s quantum path^12,27^. A good agreement is achieved between theory and experiment within the experimental error of our measurement. We note that while other electron trajectories contribute to the photoelectron momentum distribution, their respective interference patterns change rapidly in the parallel momentum direction^28^. These structures are averaged out due to intensity averaging within the many-cycle laser pulse as well as by focal volume averaging. This is in contrast with the spider interference pattern, which is relatively insensitive to shifts in the parallel momentum direction.

In order to connect the measured two-color phases to variations in the ionization times, we apply a perturbative analysis and reconstruct the variation of the ionization times of the direct electrons. The experimental Keldysh parameter is *γ* = 1 ± 0.1. Previous studies show that in this regime both the SPA and its perturbative response to the two-color field are valid^29^. The perturbative analysis provides an analytical expression for Im{*δS*(*t*_0_, *p*, *ϕ*)}, which depends on both the real and imaginary parts of *t*_0_. A careful analysis shows that Im{*δS*(*t*_0_, *p*, *ϕ*)} changes significantly with Re{*t*_0_} only, while being almost independent of Im{*t*_0_} (see Supplementary Note 5). Figure 3b shows the reconstructed ionization times (red dots) along with the predictions of the CCSFA calculation (gray line), showing a very good agreement within the experimental uncertainty. The comparison with a classical prediction that relates the ionization time (via the value of the vector potential at this time) to the measured momentum (blue dashed line) shows a striking difference. While classically, the ionization times span over a quarter of a laser cycle, quantum mechanically they are confined to a substantially narrower range. This observation is consistent with previous experiments using HHG spectroscopy^21^, and underscores the quantum nature of the interaction.

### Reconstructing the hologram dynamics

As we extend our view away from *p*_⊥_ = 0, rapid variations of the phase across the full 2D momentum plane are observed. These variations encode the dynamical properties of both arms of the hologram, i.e., the direct and scattered trajectories. According to a semi-classical analysis (see Supplementary Note 3) the two trajectories are ionized in close proximity, separated by only a few tens of attoseconds. Once they ionize, both paths interact with the laser field following very close semi-classical paths. This proximity is probed by the perturbative laser field, and leads to subtle differences in the perturbation of the action *δS*(*t*_0_, **p**, *ϕ*). Importantly, the coherent nature of the hologram enables us to identify these subtle differences with high contrast. Away from *p*_⊥_ = 0, destructive interference occurs between the direct and scattered trajectories in the dips of the spider pattern. Destructive interference provides a differential measurement, therefore subtle differences in the *t*_0_ of each trajectory result in large variations of the oscillation phase *ϕ*_opt_(*p*_||_, *p*_⊥_). Indeed, these variations are clearly observed in our experimental results.

**Fig. 4:**
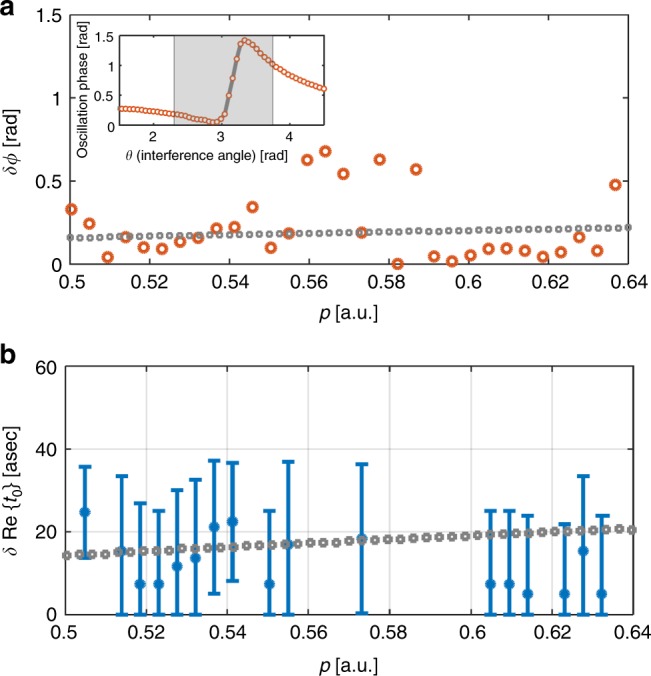
Reconstruction of the photoelectron hologram dynamics. **a** Reconstructed values for *δϕ* (red circles). The horizontal axis represents the absolute value of the momentum for which the reconstruction was performed. The CCSFA values for *δϕ* (gray squares) along the dip of the spider pattern are also shown. The experimental results and the CCSFA results are consistent and show a very minor dependence of *δϕ* on *p*. Inset: The experimentally measured two-color oscillation phase extracted along the equal energy curve (defined by $\left| {\bf{p}} \right| = 0.5$ a.u.), shown in Fig. 2b. The gray line shows the fit of our model to the experiment (red circles). The fit is performed within a narrow region around the dip (gray region). **b** Reconstructed values of the difference in ionization times, *δ*Re{*t*_0_} (blue dots). The horizontal axis is the absolute value of the momentum. The CCSFA values for *δ*Re{*t*_0_} (gray squares) along the dip of the spider pattern are also shown. Note that for the narrow momentum range 0.56a.u. < *p* < 0.59 a.u., some reconstructed *δϕ* values are beyond the range predicted by the CCSFA (due to experimental error limitations), and so the corresponding *δ*Re{*t*_0_} could not be reconstructed. An estimation of the error bars is explained in Supplementary Note 7

The perturbation by the SH field includes both a modification in the fringe’s contrast and a shift in the fringe position, as illustrated in Fig. 1. The combined effect leads to an asymmetric behavior of the two-color oscillation phase around the point of destructive interference (see Supplementary Note 6). The inset in Fig. 4a shows the oscillation phase along the equal energy curve marked by the dashed line in Fig. 2b (**|p|** = 0.5 a.u., *E* = 0.125 a.u.). Indeed, along this line a rapid and large variation of the measured phase is observed.

As described in the Supplementary Note 6, in the presence of the SH field the hologram is described by

1 $${\mathrm{Hologram}} = \left| {e^{A{\mathrm{sin}}\left( {\phi + \phi ^{\mathrm{d}}} \right)} + \rho e^{A{\mathrm{sin}}\left( {\phi + \phi ^{\mathrm{d}} + \delta \phi } \right) + iB{\mathrm{sin}}\left( {\phi + \phi ^{\mathrm{d}} + \phi ^{\mathrm{r}}} \right)}e^{i\theta }} \right|^2.$$

In this expression, *ϕ* is the relative two-color phase that is varied during the experiment, *ρ* defines the ratio of the weights of the direct and scattered electron trajectories, *θ* defines their relative phase in the absence of the SH field (and can easily be determined from a single-color hologram), *A* and *ϕ*^d^ are the amplitude and phase of the oscillation of the imaginary part of the perturbation, Im{*δS*(*t*_0_, **p**, *ϕ*)} for the direct trajectory, *δϕ* is the phase shift between the oscillation of the imaginary part of the perturbation for the direct and the scattered electrons (i.e., *δϕ* = *ϕ*^d^ − *ϕ*^s^), and finally, *B* and *ϕ*^r^ represent the amplitude and phase of the oscillation of the difference in the real part of the perturbation ΔRe(*δS*(*t*_0_, **p**, *ϕ*)), associated with a small difference in the propagation paths of the two trajectories, due to the SH field. The hologram is therefore determined by six unknown parameters: *A*, *B*, *ϕ*^d^, *δϕ*, *ϕ*^r^, and *ρ*. Within a narrow range around the dip along the equal energy curve (dashed black line in Fig. 2b), we assume that only the relative phase *θ* is modified, while all dynamical properties are constant. We fit the two-color oscillation phase response along each equal energy curve to Eq. (1) (see Supplementary Note 6). This fitting procedure enables us to reconstruct the model parameters in excellent agreement with the experiment, as shown in the inset of Fig. 4a.

## Discussion

The reconstruction procedure decouples the coherent contribution of each arm of the hologram and probes the subtle differences in their dynamical properties. We find that *ρ* ≃ 0.7 ± 0.3 along the dip of the spider pattern, reflecting a rather similar weight of the two trajectories. This differs from the *p*_⊥_ = 0 case where $\rho \gg 1$, caused by the strong dependence of *ρ* on *p*_⊥_. The subtle difference in the ionization times of the two trajectories leads to a small shift in their response to the perturbative laser field, that is encoded in *δϕ*. In Fig. 4a we show the reconstructed values of *δϕ* (orange circles), together with the theoretical prediction given by the CCSFA (gray squares)^12^. The fit and simulation are in good agreement, both showing a less than 0.5 rad difference between the response of the two trajectories for most data points. The CCSFA calculation provides us with a direct link between *δϕ* and the difference in the ionization times, *δ*Re{*t*_0_}. In the final stage of our analysis we rely on this link and reconstruct *δ*Re{*t*_0_} for each momentum value. The uncertainty in the reconstruction originates from the experimental errors in *δϕ* (see detailed analysis in the Supplementary Note 7). In Fig. 4b we show the reconstructed values of *δ*Re{*t*_0_} (blue cirlces), together with the theoretical prediction given by the CCSFA (gray squares)^12^. The fit and simulation are in good agreement, both showing a less than 50 asec interval between the ionization times of the two trajectories. Note that for the narrow momentum range 0.56 a.u. < *p* < 0.59 a.u., some reconstructed *δϕ* values are beyond the range predicted by the CCSFA (due to experimental error limitations), and so the corresponding *δ*Re{*t*_0_} could not be reconstructed.

Our study demonstrates the ability to reveal the underlying dynamics in attosecond electron holography. Focusing on *p*_⊥_ = 0, we can experimentally single out the reference arm of the hologram and reconstruct its ionization times with attosecond resolution. Studying the entire 2D momentum space, where both the reference and signal arms of the hologram are present, we are able to decouple the contributions of the two arms and reconstruct subtle differences in their ionization times. Looking forward, this approach presents a general concept which can be applied to reveal the underlying dynamics in a large range of attosecond scale strong field phenomena^30^. In many of these processes the strong laser field initiates numerous channels, while the measurement resolves their interference. Decoupling the contributions from individual channels and isolating their temporal properties will become a key component in our ability to fully resolve the complex dynamics under study. Applying our scheme at longer wavelengths will increase the dynamic range of the hologram, simplifying the interference pattern as well as allowing the investigation of higher photoelectron energies. In addition to the temporal information, the hologram contains high resolution spatial information of the interacting medium^13^. Applying our scheme in more complex systems will provide access to both spatial and temporal information currently out of reach, such as the attosecond evolution of correlated electrons in many-atom molecules and clusters.

### Data availability

The data that support the findings of this study are available from the corresponding author on request.

## Electronic supplementary material

Supplementary Information
