# Supplementary material for: Attosecond time-resolved photoelectron holography
Source: Nat Commun. 2018 Jul 18;9:2805. doi: 10.1038/s41467-018-05185-6 (PMC6051996; doi:10.1038/s41467-018-05185-6)
Supplement: Supplementary file 1 — Supplementary Information [file 41467_2018_5185_MOESM1_ESM.pdf]

Attosecond time-resolved photoelectron holography

Supplementary Information

G. Porat*[1,2], G. Alon*[2], S. Rozen[2], O. Pedatzur[2], M. Krüger[2], D. Azoury[2], A. Natan[3], G. Orenstein[2], B. D. Bruner[2], M. J. J. Vrakking[4] and N. Dudovich†[2]

[1]JILA, National Institute of Standards and Technology and University of Colorado-Boulder, Boulder, Colorado 80309-0440, USA
[2]Department of Physics of Complex Systems, Weizmann Institute of Science, 76100, Rehovot, Israel
[3]Stanford PULSE Institute, SLAC National Accelerator Laboratory, Menlo Park, California 94025, USA
[4]Max-Born-Institut, Max Born Strasse 2A, D-12489 Berlin, Germany.

June 3, 2018

Contents

Supplementary Note 1: Semi-classical approximation of the electron hologram experiment **2**

*These authors contributed equally to this work
†email:nirit.dudovich@weizmann.ac.il

Supplementary Note 2: Including the Second Harmonic field in the Semi-classical approximation **3**

Supplementary Note 3: Coulomb - corrected strong field approximation **5**

Supplementary Note 4: Numerical considerations in the CCSFA calculation **8**

Supplementary Note 5: Reconstructing the direct trajectory's ionization time **11**

Supplementary Note 6: Reconstructing the full hologram dynamics **15**

Supplementary Note 7: Reconstructing the ionization time difference between the direct and scattered electron trajectories **17**

Supplementary Note 8: Experimental set up **19**

Supplementary Note 1: Semi-classical approximation of the electron hologram experiment

In this section we introduce the semi-classical approximation that describes the strong-field interaction leading to photoelectron holography. This approximation is known as the strong-field approximation (SFA) [1, 2, 3]. The SFA describes the interaction between a ground state electron and a strong laser field. The amplitude of an electron arriving directly at the detector with final momentum **p** is a sum over all possible quantum trajectories [4, 5],

$$a_{\mathbf{p}} = -i \int_{-\infty}^{\infty} \mathrm{d}(\mathbf{p}, \mathrm{t}_0) \cdot e^{iS_{\mathbf{p}}(\mathrm{t}_0)} d\mathrm{t}_0. \tag{1}$$

where t_0 and **p** are the ionization time and final momentum of each trajectory. The dipole moment, $\mathrm{d}(\mathbf{p}, \mathrm{t}_0)$, is the matrix element between the ground and continuum states. The phase of each trajectory is given by the semi-classical action,

$$S^{\mathrm{d}}_{\mathbf{p}}(\mathrm{t}_0) = -\int_{\mathrm{t}_0}^{\infty} \frac{(\mathbf{p} - \mathbf{A}(\tau))^2}{2} d\tau - \int_{-\infty}^{\mathrm{t}_0} \mathrm{I}_{\mathrm{p}} d\tau, \tag{2}$$

where $\mathrm{I_p}$ is the ionization potential and $\mathbf{A}(\mathrm{t})$ the time-dependent vector potential of the laser field. The first term in the semi-classical action describes the propagation of a free electron in a laser field. The second term accounts for the phase gained by the electron prior to ionization.

The semi-classical expression, supplementary Eq. (2), can be modified to describe rescattered electrons as well,

$$S^{\mathrm{s}}_{\mathbf{p}}(\mathrm{t_0},\mathrm{t_1},\mathbf{k}) = -\int_{\mathrm{t_1}}^{\infty}\frac{(\mathbf{p}-\mathbf{A}(\tau))^2}{2}d\tau - \int_{\mathrm{t_0}}^{\mathrm{t_1}}\frac{(\mathbf{k}-\mathbf{A}(\tau))^2}{2}d\tau - \int_{-\infty}^{\mathrm{t_0}}\mathrm{I_p}d\tau. \quad (3)$$

Here, $\mathbf{k}$ is the drift momentum gained by the electron before scattering into the final momentum $\mathbf{p}$ and $\mathrm{t_1}$ is the moment of the scattering event. The coherent sum of the direct and scattered trajectory contributions describes the photoelectron hologram that we measure.

To evaluate these integrals, we apply a stationary phase approximation (SPA). The SPA approximates the integral using the most dominant trajectory according to

$$\nabla S_{\mathbf{p}}\big|_{\mathrm{t_0},\mathrm{t_1},\mathbf{k}} = 0. \quad (4)$$

In general, the stationary parameters can be complex. We associate the real part of $\mathrm{t_0}$ with the time at which the electron appears in the continuum, and its imaginary part with the ionization amplitude.

Supplementary Note 2: Including the Second Harmonic field in the Semi-classical approximation

In this section we add the SH field to the Semi-classical approximation. Specifically, we show that the change in the mapping between the measured photoelectron momentum value and stationary parameters arises from a higher order (i.e. beyond first order) perturbation in the second harmonic field amplitude. Since we are using a very weak SH field (0.01 by field strength), we are allowed to neglect the higher order corrections to the mapping. Therefore, the link between each momentum value and the stationary parameters does not change with the two color delay.

The modification in the semi-classical action of the direct electron can be de-

scribed as:

$$\begin{aligned} \mathrm{S}(\mathrm{t}_0,\mathbf{p}) &= \frac{1}{2}\int_{\mathrm{t}_0}^{\infty} \mathrm{p}_\perp^2 + (\mathrm{p}_{||} - \mathrm{A}_0\sin(\omega\mathrm{t}) - \epsilon\mathrm{A}_{2\omega}\sin(2\omega\mathrm{t}+\phi))^2\mathrm{dt} - \mathrm{I_p t_0} \\ &= \frac{1}{2}\int_{\mathrm{t}_0}^{\infty} \mathrm{p}_\perp^2 + (\mathrm{p}_{||} - \mathrm{A}_0\sin(\omega\mathrm{t}))^2 dt - \mathrm{I_p t_0} \\ &- \epsilon\mathrm{A}_{2\omega}\int_{\mathrm{t}_0}^{\infty} \sin(2\omega\mathrm{t}+\phi)(\mathrm{p}_{||} - \mathrm{A}_0\sin(\omega\mathrm{t}))\mathrm{dt} \\ &+ \epsilon^2\frac{\mathrm{A}_{2\omega}^2}{2}\int_{\mathrm{t}_0}^{\infty} \sin(2\omega\mathrm{t}+\phi)^2\mathrm{dt}, \end{aligned} \tag{5}$$

where $\mathrm{A}_0(\mathrm{A}_{2\omega})$ is the vector potential of the fundamental laser field (SH field) respectively, t_0 is the unperturbed ionization time, $\mathrm{I_p}$ is the ionization potential, ω is the fundamental laser frequency and ϵ is the ratio between the fundamental and SH field amplitudes. We identify the first two terms in supplementary Eq. (5) as:

$$\mathrm{S}_0(\mathrm{t}_0,\mathbf{p}) = \frac{1}{2}\int_{\mathrm{t}_0}^{\infty} \mathrm{p}_\perp^2 + (\mathrm{p}_{||} - \mathrm{A}_0\sin(\omega\mathrm{t}))^2\mathrm{dt} - \mathrm{I_p t_0} \tag{6}$$

$$\sigma(\mathrm{t}_0,\mathbf{p},\phi) = -\mathrm{A}_{2\omega}\int_{\mathrm{t}_0}^{\infty} \sin(2\omega\mathrm{t}+\phi)(\mathrm{p}_{||} - \mathrm{A}_0\sin(\omega\mathrm{t}))\mathrm{dt} \tag{7}$$

Without loss of generality, we develop the ionization time, t_0, in orders of ϵ:

$$\mathrm{t}_0 = \mathrm{t}_0^{(0)} + \epsilon\mathrm{t}_0^{(1)} + o(\epsilon^2) \tag{8}$$

The action now takes the form:

$$\mathrm{S}(\mathrm{t}_0,\mathbf{p}) = \mathrm{S}_0(\mathrm{t}_0^{(0)} + \epsilon\mathrm{t}_0^{(1)},\mathbf{p}) + \epsilon\sigma(\mathrm{t}_0^{(0)} + \epsilon\mathrm{t}_0^{(1)},\mathbf{p},\phi) + o(\epsilon^2) \tag{9}$$

Next, we derive the action to first order in ϵ:

$$\mathrm{S}(\mathrm{t}_0,\mathbf{p}) = \mathrm{S}|_{\epsilon=0} + \epsilon\frac{\mathrm{dS}}{\mathrm{d}\epsilon}|_{\epsilon=0} + o(\epsilon^2) \tag{10}$$

Each of the terms takes the following form:

$$\mathrm{S}|_{\epsilon=0} = \mathrm{S}(\mathrm{t}_0^{(0)},\mathbf{p}) \tag{11}$$

$$\epsilon \frac{dS}{d\epsilon}|_{\epsilon=0} = \epsilon\sigma(t_0^{(0)}, \mathbf{p}, \phi) - \epsilon(\frac{(p_{||} - A_0 \sin(\omega t))^2}{2} + \frac{p_\perp^2}{2} + I_p)t_0^{(1)} = \epsilon\sigma(t_0^{(0)}, \mathbf{p}, \phi) - \epsilon \frac{dS_0}{dt_0}|_{t_0^{(0)}} t_0^{(1)} \quad (12)$$

We notice that the term multiplying $t_0^{(1)}$ vanishes due to the unperturbed stationary equation. In other words, using the unperturbed stationary equation,

$$\frac{dS_0}{dt_0}|_{t_0^{(0)}} = \frac{(p_{||} - A_0 \sin(\omega t))^2}{2} + \frac{p_\perp^2}{2} + I_p = 0, \quad (13)$$

we find that that $S(t_0, \mathbf{p}) = S_0(t_0^{(0)}, \mathbf{p}) + \epsilon\sigma(t_0^{(0)}, \mathbf{p}, \phi) + o(\epsilon^2)$

We conclude that the first order correction to S, represented by σ, is not influenced by the modification to the ionization time. Such a correction contributes to higher orders in ϵ, only. A similar derivation is applied to the scattered electron.

Experimentally, we verified that the hologram is modulated according to first order perturbation. To do this, we performed a systematic study of the perturbation using different SH field intensities. Once the perturbation is reduced to a first order response, a Fourier analysis of the modulated signal shows only one spectral component - the SH frequency, 2ω. The second order response will lead to the appearance of a 4ω frequency. As can be seen in supplementary Eq. (7), seeing only the SH frequency implies that the experiment is linear in the SH field strength.

Supplementary Note 3: Coulomb - corrected strong field approximation

The Coulomb-corrected SFA is fully described in [6, 7]. The following sections 2 and 3 outline the CCSFA's procedure. The basis of the Coulomb-corrected SFA calculation is the use of **Coulomb free** saddle-point solutions in order to launch trajectories that together build the momentum map. The saddle-point solutions follow from solving the stationary equation (supplementary Eq. (4)) for t_0:

$$I_p + \frac{1}{2}(\mathbf{p} + \mathbf{A}(t_0))^2 = 0. \quad (14)$$

For a single-color laser field with a constant electric field amplitude, the saddle-point solutions can be obtained analytically. Inserting

$$\mathbf{A}(t) = -\frac{F}{\omega} \sin(\omega t) \cdot \hat{\mathbf{e}}_{||} \quad (15)$$

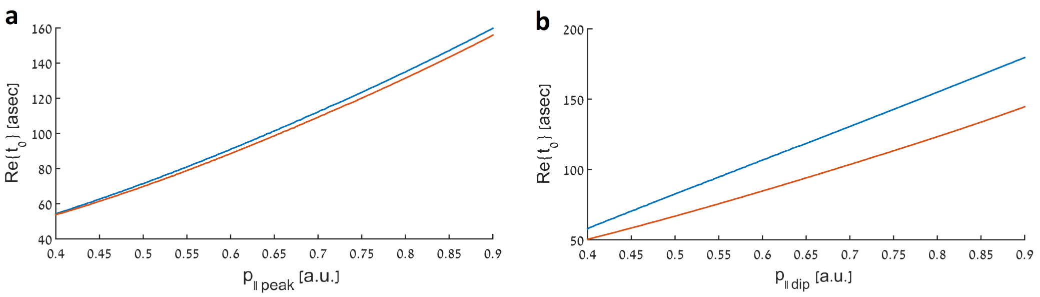

Supplementary Figure 1: The ionization times of the direct (blue) and scattered (red) electron trajectories calculated using the CCSFA (a) The ionization times along the main peak of the spider pattern, i.e. along $\mathrm{p}_\perp = 0$. $\mathrm{p}_{||\,\mathrm{peak}}$ is the parallel momentum along the main peak. (b) The ionization times calculated along the first destructive interference valley of the spider pattern. $\mathrm{p}_{||\,\mathrm{dip}}$ is the parallel momentum along the first dip.

into supplementary Eq. (14), using $\mathbf{p} = (\mathrm{p}_\perp, \mathrm{p}_{||})$, and using the fact that the laser polarization is along the parallel direction, one can write

$$\mathrm{I_p} + \frac{1}{2}\mathrm{p}_\perp{}^2 + \frac{1}{2}[\mathrm{p}_{||} + \mathrm{A}(\mathrm{t}_0)]^2 = 0, \tag{16}$$

leading to

$$\mathrm{t}_0 = \frac{1}{\omega}\arcsin(Z_1) \tag{17}$$

with

$$Z_1 = (-\mathrm{p}_{||} + i\sqrt{2\mathrm{I_p} + \mathrm{p}_\perp{}^2})\frac{\omega}{F}. \tag{18}$$

Using this result, we can calculate the ionization times, $Re\{\mathrm{t}_0\}_{\mathrm{d,s}}$, of the direct and scattered electron trajectories respectively. Supplementary figure 1a shows the ionization times of both trajectories along the main peak of the spider pattern, i.e. along $\mathrm{p}_\perp = 0$. As can be seen, the ionization time difference between the two trajectories is very small, i.e. less than 5 asec. Supplementary figure 1b shows the ionization times of the two trajectories along the first interference minimum of the spider pattern. In this case, the ionization time difference between the trajectories is larger than along the main peak, reaching a maximum of about 35 asec. The main result of our work, as reported in the main manuscript, is the

experimental determination of the times and time differences that are observable in supplementary figure 1.

In a two-color field, we again need to solve supplementary Eq. (14), but now the vector potential has the form:

$$\mathbf{A}(\mathrm{t}) = [-A_0 \sin(\omega \mathrm{t}) - \epsilon \frac{A_0}{2} \sin(2\omega \mathrm{t} + \phi)], \tag{19}$$

where ϵ is the amplitude ratio of the SH and fundamental laser fields. In this document we will refer to the phase ϕ that appears in this expression as the relative two-color phase. At all positions in the momentum map, the signal will oscillate as a function of ϕ. We will once more refer to the phase of this oscillation, i.e. the value of ϕ where the intensity is maximal, as the two-color oscillation phase, ϕ_{opt}.

In the two-color case, the saddle-point equation using the expression for the vector potential given in supplementary Eq. (19) is no longer analytically solvable, and therefore the solution of the saddle-point equation was implemented numerically using the Nelder-Mead simplex algorithm. In doing so, supplementary Eq. (17) was used as the starting point for the search, which works well since the SH field is only a small perturbation.

In the Coulomb-corrected SFA one numerically solves trajectories starting from the saddle-point solution. The equations of motion are:

$$\frac{d^2}{dt^2}\mathbf{r} = \frac{d}{dt}\mathbf{v} = (-\mathbf{E}(\mathrm{t})) \cdot \hat{\mathbf{e}}_{||} - \frac{1}{\mathrm{r}^2} \cdot \hat{\mathbf{e}}_{\perp}, \tag{20}$$

where **r** and **v** are the electron's position and velocity respectively, and $\mathbf{E}(\mathrm{t})$ is the laser's electric field. In order to calculate the trajectories, a choice has to be made for the initial velocity $\mathbf{v}(\mathrm{t}_0)$ and the initial position $\mathbf{r}(\mathrm{t}_0)$. The initial velocity at the saddle-point is chosen by demanding that - in a calculation without inclusion of the Coulomb potential - the final measured momentum (after the laser pulse is over) is the chosen canonical momentum $\mathbf{p} = \mathbf{v}(\mathrm{t}) - \mathbf{A}(\mathrm{t})$. i.e.

$$\mathbf{v}(\mathrm{t}_0) = \mathbf{p} + \mathbf{A}(\mathrm{t}_0). \tag{21}$$

The initial position $\mathbf{r}(\mathrm{t}_0)$ is chosen by requiring that (i) the real part of the trajectory starts at the origin (i.e $\mathbf{r}(\mathrm{t}_0)$ is purely imaginary), and (ii) the imaginary part of the trajectory vanishes when the tunneling process has been completed (i.e. when the integration over imaginary time is completed).

The amplitude that a particular trajectory contributes to the momentum map is equal (up to a number of momentum-independent pre-factors) to

$$\mathrm{F}_{\mathbf{p},\mathrm{t}_0} \simeq \sqrt{\frac{1}{S''(\mathbf{p},\mathrm{t}_0)}} e^{iS(\mathbf{p},\mathrm{t}_0)}. \tag{22}$$

In the calculation, $S(\mathbf{p},\mathrm{t}_0)$ is numerically integrated along the trajectory. During the imaginary time integration, the integral

$$S_1 = \int_{\mathrm{t}_0}^{Re\{\mathrm{t}_0\}} dt'[\frac{1}{2}(\mathbf{p}+\mathbf{A}(t'))^2 + \mathrm{I_p}] = \int_{\mathrm{t}_0}^{Re\{\mathrm{t}_0\}} dt'[\frac{1}{2}{v_\perp}^2 + \frac{1}{2}v_{||}(t')^2 + \mathrm{I_p}], \tag{23}$$

is evaluated. When the real time propagation starts, the Coulomb potential is turned on, and the integral is evaluated as

$$S_2 = \int_{Re\{\mathrm{t}_0\}}^{\infty} dt'[\frac{1}{2}{v_\perp}^2 + \frac{1}{2}v_{||}(t')^2 + \mathrm{I_p} - \frac{1}{r}]. \tag{24}$$

The second derivative of the action at the saddle-point, $S^{''}(\mathbf{p},\mathrm{t}_0)$, is calculated by taking the 2nd derivative of supplementary Eq. (23), leading to

$$S^{''}(\mathbf{p},\mathrm{t}_0) = (\mathbf{p}+\mathbf{A}(\mathrm{t}_0)) \cdot \frac{d\mathbf{A}(\mathrm{t})}{dt}(\mathrm{t}_0) = -Im(\mathbf{A}(\mathrm{t}_0)\cdot\mathbf{E}(\mathrm{t}_0)). \tag{25}$$

Supplementary Note 4: Numerical considerations in the CCSFA calculation

The above equations are implemented in a program that calculates the trajectories for a set of initial conditions that are derived from the requirement that the final canonical momentum spans a large enough grid. In practice, calculations were run for

$$\mathrm{p}_{||} = (-10000.....10000) \times 0.00016\,\mathrm{a.u.}$$

$$\mathrm{p}_{\perp} = (-6000.....0) \times 0.00016\,\mathrm{a.u.} \tag{26}$$

Trajectories calculated for $\mathrm{p}_\perp < 0$ mirror trajectories that are calculated for $\mathrm{p}_\perp > 0$. Therefore, only trajectories with $\mathrm{p}_\perp \leq 0$ are calculated, and all results are used

twice, flipping the sign of the final outgoing momentum. Mimicking the experimental conditions and ensuring that the SH field was a sufficiently weak perturbation, calculations were performed for $\omega_{\mathrm{IR}} = 0.0579\,\mathrm{a.u.}$, $\mathrm{F_{IR}} = 0.0609\,\mathrm{a.u.}$, $\mathrm{I_p} = 0.5790\,\mathrm{a.u.}$ and $\mathrm{F_{SH}} = 0.0006\,\mathrm{a.u.}$ A laser field was used where all ionization events took place during a single cycle and where the amplitude of the laser field was constant, followed by a six-cycle cos-squared turn-off of the field.

A calculation as specified takes about 3 days on a single processor for a single two-color delay. However, while that gives good enough statistics for final momenta where the direct and the indirect path interfere constructively, this still leads to very poor statistics in the valleys of the photoelectron hologram where the direct and the indirect path interfere destructively. To overcome this, an interpolation method was implemented to generate large numbers of additional trajectories on the basis of the numerically calculated trajectories. An interpolation was implemented where at each location calculations were considered using the following 5 canonical momenta as input:

$$(\mathrm{p}_{||} + \delta\mathrm{p}, \mathrm{p}_{\perp}), (\mathrm{p}_{||}, \mathrm{p}_{\perp} - \delta\mathrm{p}), (\mathrm{p}_{||}, \mathrm{p}_{\perp}), (\mathrm{p}_{||}, \mathrm{p}_{\perp} + \delta\mathrm{p}), (\mathrm{p}_{||} - \delta\mathrm{p}, \mathrm{p}_{\perp}). \tag{27}$$

The trajectory calculations for these 5 cases were used to generate N trajectories by means of a quadratic interpolation, with the input canonical momentum in the range $(\mathrm{p}_{||} \pm \frac{1}{2}\delta\mathrm{p}, \mathrm{p}_{\perp} \pm \frac{1}{2}\delta\mathrm{p})$, and with N typically 100 or 300. A momentum map was calculated using all these trajectories. Even more importantly, while generating this momentum map, all the trajectories leading to a particular final detected momentum were grouped into sets on the basis of the similarity of their input, i.e. the (Coulomb-free) canonical momentum $(\mathrm{p}_{||-\mathrm{start}}, \mathrm{p}_{\perp-\mathrm{start}})$, as well as the similarity of the accumulated action along the trajectory. So the total number of trajectories leading to a particular final momentum (i.e a particular pixel in the momentum map), was grouped into sets of qualitatively distinct trajectories, where (in particular after filtering out cases where re-collisions had an impact parameter of $\mathrm{R_{min}} \leq 1.5\,\mathrm{a.u.}$ or a very late closest approach of the electron to the ion core after ionization, $\mathrm{T_{min}} \geq 150\,\mathrm{a.u.}$) the most frequently occurring trajectory ($\mathrm{N_1}$ times) would typically correspond to the direct trajectory, and the next-most frequently occurring trajectory ($\mathrm{N_2}$ times) would be the rescattered trajectory. As a result of this procedure, the contribution of one of the sets to the momentum map is given as

$$\mathrm{F}_{\mathbf{p},\mathrm{t}_0} \simeq \mathrm{N_i}\sqrt{\frac{1}{S''(\mathbf{p},\mathrm{t}_0)_{avg}}}\, e^{iS(\mathbf{p},\mathrm{t}_0)_{avg}}, \tag{28}$$

where the averages are evaluated by considering all the individual trajectories that contribute to the set. The latter corresponds to making the following approximation for all the trajectories j that have been selected to be part of a particular set

$$\sum_j \sqrt{\frac{1}{S''(\mathbf{p},\mathrm{t}_0)_j}}\, e^{iS(\mathbf{p},\mathrm{t}_0)_j} \sim \mathrm{N_i} \sqrt{\frac{1}{S''(\mathbf{p},\mathrm{t}_0)_{\mathrm{avg}}}}\, e^{iS(\mathbf{p},\mathrm{t}_0)_{\mathrm{avg}}}. \tag{29}$$

While calculating a momentum map on the basis of the leading sets (N_1 and N_2) led to aesthetically pleasing momentum maps, plots of the phase of the two-color oscillation were still more noisy (especially in the valleys) than desirable. Therefore, two further types of averaging were implemented. Prior to their use to evaluate the momentum map, the variables determining the amplitude (i.e $\mathrm{N}_i, S, \mathbf{A}(\mathrm{t}_0), \mathbf{E}(\mathrm{t}_0)$) were subjected to (5x5) point Savitsky-Golay filtering. Furthermore, when calculating the oscillation of the calculated intensity at a particular final momentum as a function of the relative two-color phase ϕ, the results were also averaged over (5x5) pixels, with the chosen final momentum at the center.

The afore-mentioned grouping of the trajectories in the course of the interpolation, is necessary to make the further analysis and processing manageable. In practice we could perform these operations for a 400x400 grid covering $0 \leq \mathrm{p}_\perp \leq 1.0\,\mathrm{a.u.}$ and $0 \leq \mathrm{p}_{||} \leq 1.0\,\mathrm{a.u.}$ The 400x400 grid led to some undersampling (aliasing) in the calculated momentum map. To alleviate this, we fitted the action to a quadratic function of $\mathrm{p}_{||}$ for each value of $\mathrm{p}_{||}$ (which gives very good results, except for $\mathrm{p}_{||} < 0.2\,\mathrm{a.u.}$, where the dependence of the action on $\mathrm{p}_{||}$ is more complicated; however, our main interest will presently be in an analysis for $\mathrm{p}_{||} > 0.2\,\mathrm{a.u.}$).

Supplementary figure 2 shows four ways that, on the basis of the above explanation, the momentum map can be calculated. First, a map can be directly obtained on the basis of the trajectories that are calculated (supplementary figure 2a). This map contains both the direct and indirect trajectories that are responsible for the holographic interference, as well as a large variety of more complicated trajectories that result from delayed re-collisions (i.e. late returns). These can be suppressed, e.g. by requiring that after tunneling the electron is not allowed to approach the ion within a radius $\mathrm{R}_{\mathrm{min}}$ (typically $1.5\,\mathrm{a.u.}$) or by requiring that the closest approach has to occur within a time $\mathrm{T}_{\mathrm{min}}$ (typically $150\,\mathrm{a.u.}$). A map where these requirements are implemented is shown in supplementary figure 2b. As explained before, the number of trajectories resulting from the calculations is insufficient for a satisfactory determination of the two-color phase dependence at

all final momenta. Therefore an interpolation is done, resulting in a sorting of all trajectories that produce a given final momentum (i.e. for a given pixel in the momentum map), on the basis of the similarity of their input (i.e. the similarity of the initial (Coulomb-free) canonical momentum and the similarity of the accumulated action along the trajectory). For all members of these sets, the average (Coulomb-free) canonical momentum, the number of members in the set and the calculated classical action are stored for later use in supplementary Eq. (28). These grouped trajectories can be used to again calculate the momentum map (with or without the inclusion of re-collision trajectories with $\mathrm{R_{min}} < 1.5\,\mathrm{a.u.}$ and/or $\mathrm{T_{min}} > \mathrm{a.u.}$). Supplementary figure 2c shows the result when these trajectories are excluded. The result shown in supplementary figures 2a and b is largely reproduced, but one can clearly see the improved signal-to-noise, at the expense of some aliasing resulting from the fact that the 400x400 grid that is used here is necessarily a bit cruder. Correspondingly, it is helpful to sort the sets so as to isolate the direct and indirect trajectories that give rise to the holographic pattern, and then fit the calculated (and interpolated) action of these contributions to a quadratic function of $\mathrm{p}_{||}$ for each value of $\mathrm{p}_{||}$. This gives rise to the momentum map that is shown in supplementary figure 2d. The intensity values in this plot permit the highest quality determination of the phase of the two-color oscillation ϕ_{opt}, and manage to recover this phase successfully even in the ”valleys” of the momentum map where the holographic interference is destructive. Comparing supplementary figure 2d with the experimental momentum map (figure 2a of the main text) we find a good agreement in the structure of the hologram, both in the energy and separation of the holographic fringes.

Supplementary Note 5: Reconstructing the direct trajectory’s ionization time

Along $\mathrm{p}_{\perp} = 0$, the direct and scattered electron trajectories interfere constructively, encoding their average response. Since their ionization times are very similar (see Supplementary figure 1), the perturbation has a similar effect on both (direct and scattered) amplitudes. Due to scattering, the amplitude of the direct electron trajectory is substantially greater than that of the scattered electron trajectory. Therefore, the average response can be approximated as simply the direct trajectory response.

We aim to use the CCSFA in order to reconstruct the ionization times of the

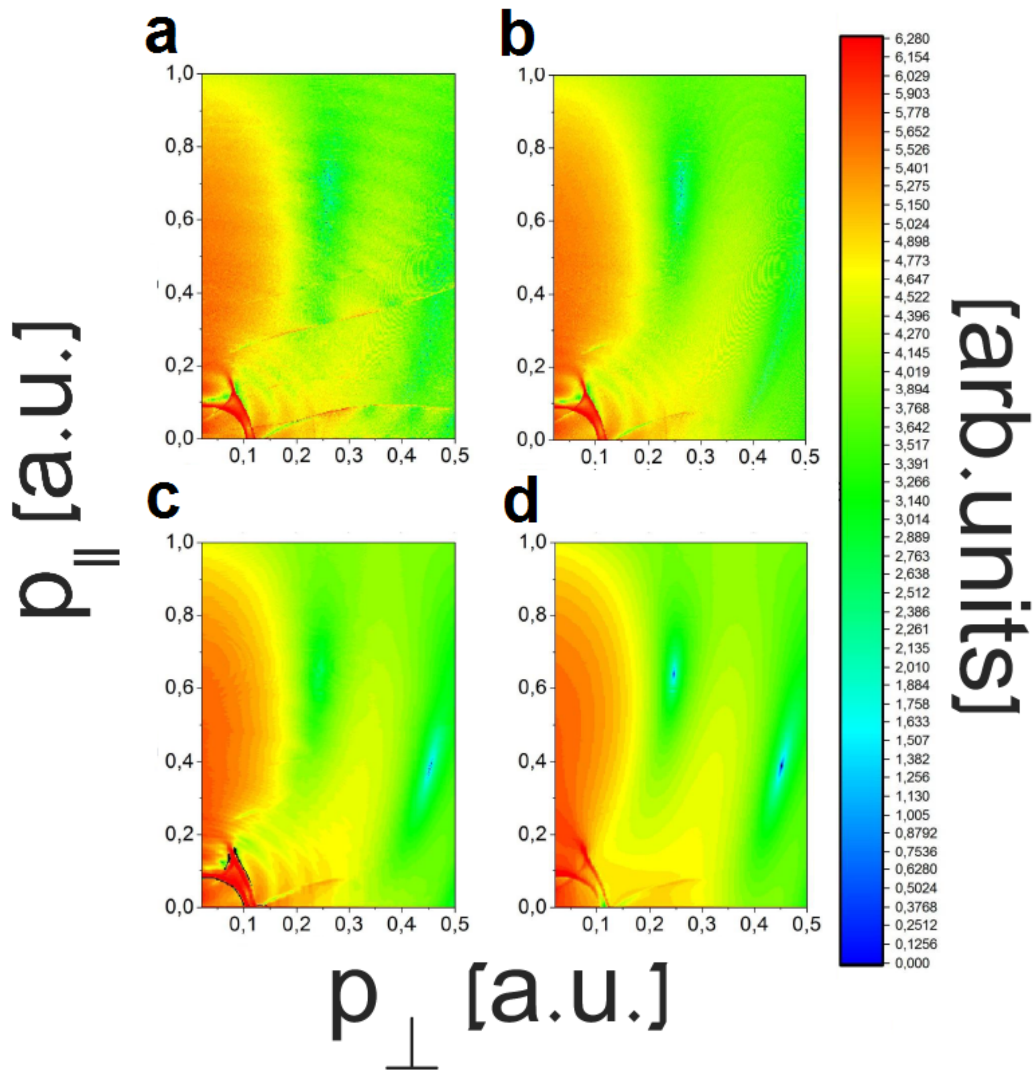

Supplementary Figure 2: Momentum maps for two-color ionization of Ar using the parameters specified in the text (a) Momentum map on the basis of the calculated trajectories; (b) idem, but suppressing trajectories where $\mathrm{R_{min}} < 1.5\,\mathrm{a.u.}$ or $\mathrm{T_{min}} > 150\,\mathrm{a.u.}$; (c) momentum map after 300-fold enhancement of the number of trajectories by interpolation, while suppressing trajectories where $\mathrm{R_{min}} < 1.5\,\mathrm{a.u.}$ or $\mathrm{T_{min}} > 150\,\mathrm{a.u.}$; (d) idem, retaining only the contributions of the dominant direct and indirect trajectories that give rise to the holographic pattern, after fitting the classical action and Savitsky-Golay filtering. All plots are plotted on a logarithmic color scale that covers 6 orders of magnitude

direct electron trajectory. With only one dominant contribution from the direct trajectory, the measurement is sensitive to amplitude modulations only

$$\mathrm{I}^{\mathrm{d}} = |e^{iS^{\mathrm{d}}(\mathbf{p},\mathrm{t}_0)}|^2 = e^{-2\mathrm{Im}\{S^{\mathrm{d}}(\mathbf{p},\mathrm{t}_0)\}}, \tag{30}$$

where the superscript "d" is used to indicate that $S(\mathbf{p},\mathrm{t}_0)$ refers to the direct trajectory. The imaginary contribution to the perturbed action, supplementary Eq. (23), is associated with the integration along the imaginary components of t_0 [5]

$$\mathrm{Im}(S^{\mathrm{d}}(\mathbf{p},\mathrm{t}_0)) = \mathrm{Im}(\int_{Re\{\mathrm{t}_0\}+i\mathrm{Im}\{\mathrm{t}_0\}}^{\mathrm{Re}\{\mathrm{t}_0\}} [\mathrm{p}_\perp^2 + (\mathrm{p}_{||} - \mathrm{A}_0\mathrm{sin}(\omega t) - \frac{\mathrm{A}_0}{2}\epsilon\sin(2\omega t+\phi))^2 + \mathrm{I}_\mathrm{p}]\, dt), \tag{31}$$

where $\epsilon \ll 1$ is the ratio of the second harmonic and fundamental fields [5]. Since the SH field is perturbative, we expand the action to first order in ϵ. The first order terms are:

$$\begin{aligned}\left.\frac{\partial\mathrm{Im}\{S^{\mathrm{d}}(\mathbf{p},\mathrm{t}_0)\}}{\partial\epsilon}\right|_{\epsilon=0} = \frac{1}{2}\mathrm{Im}\{&\frac{\mathrm{A}_0\mathrm{p}_{||}\mathrm{cos}(\phi+2\omega\mathrm{Re}\{\mathrm{t}_0\})}{\omega} - \frac{\mathrm{A}_0\mathrm{p}_{||}\mathrm{cos}(\phi+i2\omega\mathrm{Im}\{\mathrm{t}_0\}+2\omega\mathrm{Re}\{\mathrm{t}_0\})}{\omega}\\ &+\frac{\mathrm{A}_0^2\mathrm{sin}(\phi+\omega\mathrm{Re}\{\mathrm{t}_0\})}{\omega} - \frac{\mathrm{A}_0^2\mathrm{sin}(\phi+i\omega\mathrm{Im}\{\mathrm{t}_0\}+\omega\mathrm{Re}\{\mathrm{t}_0\})}{\omega}\\ &-\frac{\mathrm{A}_0^2\mathrm{sin}(\phi+3\omega\mathrm{Re}\{\mathrm{t}_0\})}{3\omega} + \frac{\mathrm{A}_0^2\mathrm{sin}(\phi+i3\omega\mathrm{Im}\{\mathrm{t}_0\}+3\omega\mathrm{Re}\{\mathrm{t}_0\})}{3\omega}\}.\end{aligned} \tag{32}$$

Taking the imaginary part provides

$$\begin{aligned}\left.\frac{\partial\mathrm{Im}\{S^{\mathrm{d}}(\mathbf{p},\mathrm{t}_0)\}}{\partial\epsilon}\right|_{\epsilon=0} &= -\frac{\mathrm{A}_0^2\mathrm{cos}(\phi+\omega\mathrm{Re}\{\mathrm{t}_0\})\mathrm{sinh}(\omega\mathrm{Im}\{\mathrm{t}_0\})}{2\omega}\\ &-\frac{\mathrm{A}_0\mathrm{p}_{||}\mathrm{sin}(\phi+2\omega\mathrm{Re}\{\mathrm{t}_0\})\mathrm{sinh}(2\omega\mathrm{Im}\{\mathrm{t}_0\})}{2\omega}\\ &+\frac{\mathrm{A}_0^2\mathrm{cos}(\phi+3\omega\mathrm{Re}\{\mathrm{t}_0\})\mathrm{sinh}(3\omega\mathrm{Im}\{\mathrm{t}_0\})}{6\omega}.\end{aligned} \tag{33}$$

The sum of the three terms in supplementary Eq. (33) is a single oscillating term with phase $\psi_{\mathrm{p}_{||}}$,

$$\left.\frac{\partial\mathrm{Im}\{S^{\mathrm{d}}_{\mathbf{p}}\}}{\partial\epsilon}\right|_{\epsilon=0} \sim \sin(\phi+\psi_{\mathrm{p}_{||}}). \tag{34}$$

Note that $\mathrm{Re}\{\mathrm{t}_0\}$ appears in a trigonometric function while $\mathrm{Im}\{\mathrm{t}_0\}$ in a hyperbolic trigonometric function. For $\mathrm{Im}\{\mathrm{t}_0\} \ll \frac{2\pi}{\omega}$ as indicated by the CCSFA, the

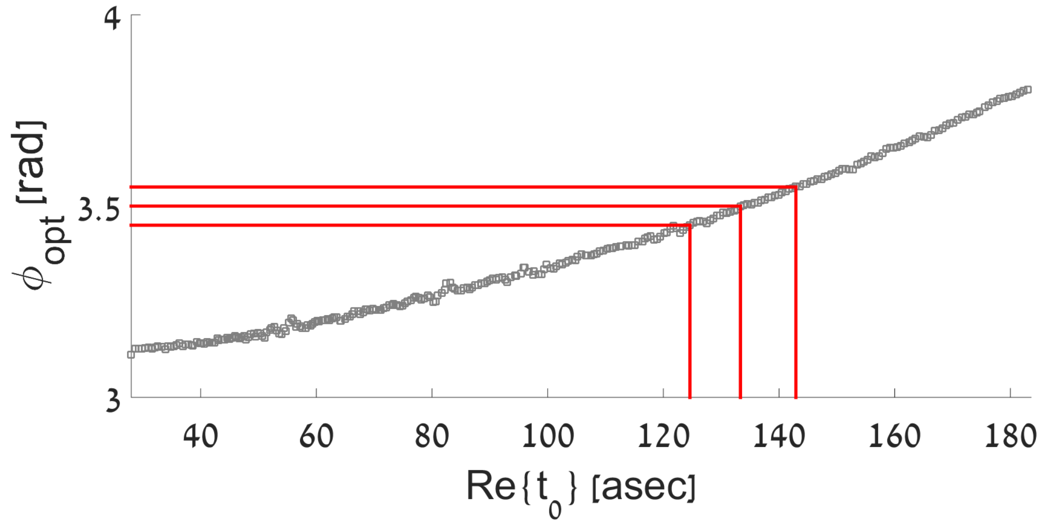

Supplementary Figure 3: The mapping between $\phi_{\rm opt}$ and $Re\{{\rm t}_0\}$ as given by the CCSFA. The CCSFA's mapping is calculated along ${\rm p}_\perp = 0.025\,{\rm a.u.}$ in the range $0.25\,{\rm a.u.} < {\rm p}_{||} < 1\,{\rm a.u.}$ The red lines show an example of our reconstruction method, as elaborated in the text. The gray squares are the CCSFA's data.

variation in the hyperbolic trigonometric function is much slower than that of the trigonometric function. Thus the dependence on ${\rm Im}\{{\rm t}_0\}$ is significantly weaker.

Using the CCSFA, we plot the mapping between $\phi_{\rm opt}$ and the direct trajectory's ionization time, denoted by $Re\{{\rm t}_0\}$. This mapping is shown in supplementary figure (3).

Comparing the measured $\phi_{\rm opt}$ (see figure 3a of the main text) with this mapping provides us with the average value of the reconstructed $Re\{{\rm t}_0\}$ shown in figure 3b of the main text. Supplementary figure 3 shows an example of how we determine the average $Re\{{\rm t}_0\}$. As can be seen in the figure (middle red line), and using the relationship provided by the CCSFA, we map $\phi_{\rm opt} = 3.5$ rad to a $Re\{{\rm t}_0\} \simeq 130$ asec. In the experiment, $\psi^{\rm exp}_{{\rm p}_{||}}$ is known up to a global phase which is calibrated using the CCSFA model.

Since ${\rm Re}\{{\rm t}_0\}$ is a function of three parameters, ${\rm Im}\{{\rm t}_0\}$, $\psi^{\rm exp}_{{\rm p}_{||}}$ and the global phase, the error in the reconstruction arises from three contributions. The first comes from the uncertainty in experimentally evaluating $\psi^{\rm exp}_{{\rm p}_{||}}$. The second is the use of the CCSFA value of ${\rm Im}\{{\rm t}_0\}$. The third comes from the error in assigning the global phase. To estimate the error in the reconstructed ionization time arising from the uncertainty in experimentally evaluating $\psi^{\rm exp}_{{\rm p}_{||}}$, we repeat the calculation

of $\mathrm{Re}\{\mathrm{t}_0\}$ for all $\psi^{\mathrm{exp}}_{\mathrm{p}_{||}}$ values within a standard deviation from the measured value. To estimate the error in the reconstructed ionization time arising from the use of the CCSFA value of $\mathrm{Im}\{\mathrm{t}_0\}$, we replace in supplementary Eq. (33) $\mathrm{Im}\{\mathrm{t}_0\}$ by its average value given by the CCSFA. We then equate the analytical expression for $\psi_{\mathrm{p}_{||}}$ with the experimental two-color phase, $\psi^{\mathrm{theory}}_{\mathrm{p}_{||}} = \psi^{\mathrm{exp}}_{\mathrm{p}_{||}}$, and numerically solve for $\mathrm{Re}\{\mathrm{t}_0\}$ for all $\mathrm{p}_{||}$ values and repeat this calculation of $\mathrm{Re}\{\mathrm{t}_0\}$ for all values of $\mathrm{Im}\{\mathrm{t}_0\}$ within the dynamic range predicted by the CCSFA. To estimate the error arising from the use of the CCSFA's global phase, we repeat the mapping procedure with a global phase shifted by the experimental uncertainty in evaluating $\psi^{\mathrm{exp}}_{\mathrm{p}_{||}}$. The error bars in figure 3b of the main text reflect the cummulative estimation of the error.

Figure 3b of the main text shows the reconstructed $\mathrm{Re}\{\mathrm{t}_0\}$ along with the CCSFA's $\mathrm{Re}\{\mathrm{t}_0\}$. As can be seen, the results are in excellent agreement.

Supplementary Note 6: Reconstructing the full hologram dynamics

The two-color phase map shows a distinctive asymmetric line shape around points of destructive interference (see the inset in Fig. 4a of the main text). The goal of this section is to present a simple model which describes the origin of this asymmetry, and to describe the reconstruction procedure, based on this model, of the hologram dynamics.

Using a single-color driving field, the photoelectron hologram can be described semi-classically as the coherent interferogram of two electronic trajectories, the direct and scattered electron trajectories (see section 1). The hologram is modeled using the unperturbed quasi-classical action of the two trajectories

$$\mathrm{Hologram} = \left| e^{iS^{\mathrm{d}}_{\mathbf{p}}} + e^{iS^{\mathrm{s}}_{\mathbf{p}}} \right|^2, \tag{35}$$

where we defined $S^{\mathrm{d}}(\mathbf{p}, \mathrm{t}_0) = S^{\mathrm{d}}_{\mathbf{p}}$. Adding a perturbative SH field ($\epsilon \sim 0.01$ in our experiment), we expand the action to first order in ϵ

$$S_{\mathbf{p}} \sim S_{\mathbf{p},0} + \epsilon \frac{\partial}{\partial \epsilon} S_{\mathbf{p}}. \tag{36}$$

From here we find

$$\mathrm{Hologram} \sim |e^{i[S^{\mathrm{d}}_{\mathbf{p},0} + \epsilon \frac{\partial}{\partial \epsilon} S^{\mathrm{d}}_{\mathbf{p}}]} + e^{i[S^{\mathrm{s}}_{\mathbf{p},0} + \epsilon \frac{\partial}{\partial \epsilon} S^{\mathrm{s}}_{\mathbf{p}}]}|^2. \tag{37}$$

Since the oscillation phase of each pixel of the hologram is unaffected by multiplication of both trajectory amplitudes by a global constant, the phase map is only sensitive to the ratio between the unperturbed amplitudes $\rho = \frac{e^{ImS^{\mathrm{d}}_{\mathbf{p},0}}}{e^{ImS^{\mathrm{s}}_{\mathbf{p},0}}}$. Since the hologram is sensitive to the relative phase between the two arms it can be described as:

$$\text{Hologram} = |e^{-\epsilon \mathrm{Im}\{\frac{\partial}{\partial\epsilon}S^{\mathrm{d}}_{\mathbf{p}}\}} + \rho e^{i[\mathrm{Re}\{S^{\mathrm{s}}_{\mathbf{p},0}\}-\mathrm{Re}\{S^{\mathrm{d}}_{\mathbf{p},0}\}]}e^{i\epsilon[\mathrm{Re}\{\frac{\partial}{\partial\epsilon}S^{\mathrm{s}}_{\mathbf{p}}\}-\mathrm{Re}\{\frac{\partial}{\partial\epsilon}S^{\mathrm{d}}_{\mathbf{p}}\}]}e^{-\epsilon \mathrm{Im}\{\frac{\partial}{\partial\epsilon}S^{\mathrm{s}}_{\mathbf{p}}\}}|^2 \quad (38)$$

Due to the perturbation, the imaginary part of the first order of the action oscillates as

$$-\epsilon\frac{\partial}{\partial\epsilon}\mathrm{Im}\{S^{\mathrm{d,s}}_{\mathbf{p}}\} = \mathrm{A}\sin(\phi+\phi^{\mathrm{d,s}}), \quad (39)$$

where A is the amplitude of the imaginary oscillation, which is assumed to be the same for the reference (direct) and the signal (scattering) trajectories, and $\phi^{\mathrm{d,s}}$ is the phase of the oscillation of the reference and signal trajectories respectively. The difference in the real perturbation oscillates as

$$\epsilon(\mathrm{Re}\{\frac{\partial}{\partial\epsilon}S^{\mathrm{s}}_{\mathbf{p},0}\} - \mathrm{Re}\{\frac{\partial}{\partial\epsilon}S^{\mathrm{d}}_{\mathbf{p},0}\}) = \mathrm{B}\sin(\phi+\phi^{\mathrm{d}}+\phi^{\mathrm{r}})), \quad (40)$$

where B is the amplitude of the real oscillation and ϕ^{r} is the phase of the real oscillation with respect to ϕ^{d}. Substituting this expression and defining the unperturbed interference angle as $Re\{S^{\mathrm{s}}_{\mathbf{p},0}\} - Re\{S^{\mathrm{d}}_{\mathbf{p},0}\} = \theta$ and $\delta\phi = \phi^{\mathrm{s}} - \phi^{\mathrm{d}}$, we arrive at the expression:

$$\text{Hologram} = |e^{\mathrm{A}\sin(\phi+\phi^{\mathrm{d}})} + \rho e^{\mathrm{A}\sin(\phi+\phi^{\mathrm{d}}+\delta\phi)}e^{i\mathrm{B}\sin(\phi+\phi^{\mathrm{d}}+\phi^{\mathrm{r}})}e^{i\theta}|^2, \quad (41)$$

described in eq.2 of the main text. As is shown in Fig. 4a of the main text, this model describes the variation of the two-color oscillation phase with θ in the equal energy line shapes very accurately.

The physical interpretation of the different parameters appearing in the model are as follows: $\delta\phi$ is the two-color phase response difference of the ionization amplitude between the two trajectories, ρ is the amplitude ratio of the unperturbed trajectories and A is the amplitude of the perturbation to the ionization amplitude of the direct and scattered trajectories. As stated already, we assume that this amplitude is similar for the two trajectories since they are born within less than 35 asec of each other (see supplementary figure (1)). B is the amplitude of the perturbation on the trajectories while they are in the continuum and ϕ^{r} is its phase.

Since we are looking at a narrow region around the dip of the interference pattern (see Fig. 4a of the main text), we assume that these dynamical parameters are constant for each equal energy curve.

We extract the model's parameters by fitting the model to the experimental two-color phase as follows. For a given equal energy curve and a set of model parameters $(\mathrm{A}, \mathrm{B}, \rho, \delta\phi$ and $\phi^{\mathrm{r}})$, we numerically sweep the two-color delay (ϕ) and determine the two-color phase response, ϕ_{opt}, for each pixel along the equal energy curve. The fitting procedure finds the optimal model parameters which minimize the least square error between the numerical and experimental two-color phases along all the pixels on an equal energy curve. We find excellent agreement between the model and experiment as is shown in the inset of Fig. 4a of the main text. We find that for energies of 0.12-0.24 a.u., the dynamical parameters are constant with mean values $\mathrm{A} = 0.07 \pm 0.1$, $\mathrm{B} = 0.11 \pm 0.1$ and $\phi^{\mathrm{r}} \simeq 1 \pm 0.4$ rad. These results are consistent with our model assuming perturbative amplitudes, $\mathrm{A}, \mathrm{B} \ll 1$.

Supplementary Note 7: Reconstructing the ionization time difference between the direct and scattered electron trajectories

Using the CCSFA, we plot the mapping between $\delta\phi$ and the direct and scattered trajectories' ionization time difference, denoted by $\Delta Re\{\mathrm{t}_0\}$. This mapping is shown in Fig. (4). The error bars (shaded region in Fig. 4) are ± 0.01.

Comparing the $\delta\phi$ we reconstructed from the experiment (see Fig. 4a of the main text) with this mapping provides us with the average value of the reconstructed $\Delta Re\{\mathrm{t}_0\}$ shown in Fig. 4b of the main text. Figure 4 shows an example of how we determine the average $\Delta Re\{\mathrm{t}_0\}$. As can be seen in the figure (middle red line), and using the relationship provided by the CCSFA, we map $\delta\phi = 0.17$ rad to a $\Delta Re\{\mathrm{t}_0\} \simeq 15$ asec.

There are three sources of error which contribute to the uncertainty in the reconstructed $\Delta Re\{\mathrm{t}_0\}$. The first is the error in the reconstruction of $\delta\phi$, i.e. the accuracy of our fit, which is bounded by 0.1 rad. The second is the error due to the numerical simulation in the CCSFA. The third is a result of the fact that $\delta\phi$ is a function of other stationary parameters in addition to $\Delta Re\{\mathrm{t}_0\}$. Specifically, $\delta\phi$ is also a function of the imaginary ionization times of both trajectories and the complex scattering time of the scattered trajectory. The error in this case arises from

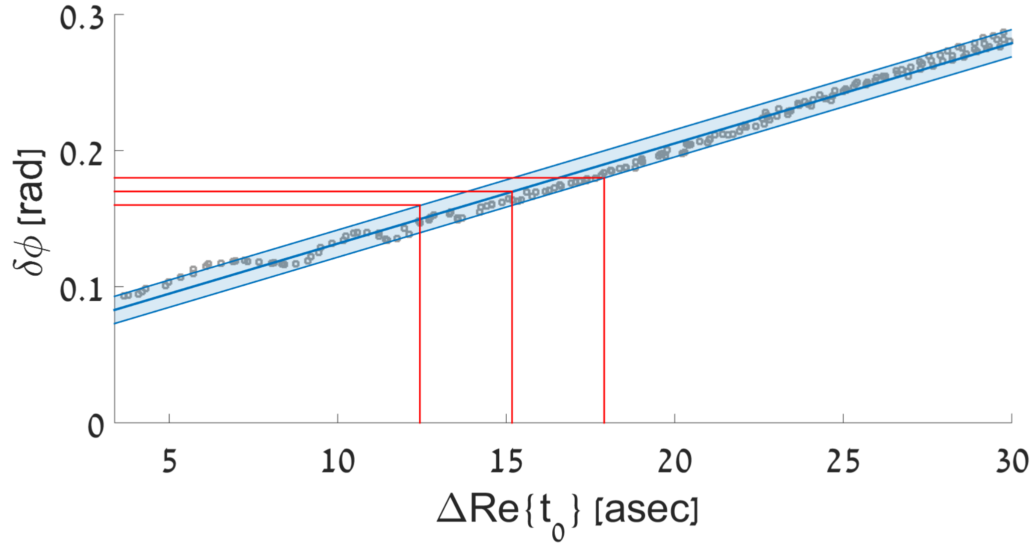

Supplementary Figure 4: The mapping between $\delta\phi$ and $\Delta Re\{\mathrm{t}_0\}$ as given by the CCSFA. The CCSFA's mapping is calculated along the first dip of the spider pattern in the range $0.36\,\mathrm{a.u.} < \mathrm{p}_{||} < 0.85\,\mathrm{a.u.}$ The error bars are ± 0.01 rad (shaded region). The red lines show an example of our reconstruction method, as elaborated in the text. The gray squares are the CCSFA's data.

allowing these stationary parameters to vary along their dynamic range, changing the mapping between $\delta\phi$ and $\Delta Re\{\mathrm{t}_0\}$. We calculate the effect of the first two error causes by mapping the upper and lower bound for the reconstructed $\delta\phi$ to the lower and upper bound of the mapping in the CCSFA, respectively. An example is shown in Fig. (4). In this figure, we map the upper and lower bound of the reconstructed $\delta\phi$ (upper and lower red lines), i.e. $\delta\phi = 0.17 \pm 0.01$ rad, to the lower and upper bound of the CCSFA's mapping respectively. For ease of illustration, Fig. 4 shows the reconstruction procedure for $\delta\phi = 0.17 \pm 0.01$ rad. To estimate the error resulting from the third cause, we use the analytic equation for $\delta\phi$ given by the SFA. This equation gives us the explicit dependence of $\delta\phi$ on each of the stationary parameters. We calculate the error as

$$\Delta\delta\phi = \sqrt{\left(\frac{\partial\delta\phi}{\partial\mathrm{Im}\{\mathrm{t}_0^{\mathrm{d}}\}}\Delta\mathrm{Im}\{\mathrm{t}_0^{\mathrm{d}}\}\right)^2 + \left(\frac{\partial\delta\phi}{\partial\mathrm{Im}\{\mathrm{t}_0^{\mathrm{s}}\}}\Delta\mathrm{Im}\{\mathrm{t}_0^{\mathrm{s}}\}\right)^2 + ...}, \tag{42}$$

where $\Delta\delta\phi$ is the error in $\delta\phi$, $\mathrm{t}_0^{\mathrm{d}}$ is the complex ionization time of the direct electron trajectory, $\mathrm{t}_0^{\mathrm{s}}$ is the complex ionization time of the scattered electron trajectory and $\Delta Im\{\mathrm{t}_0^{\mathrm{d,s}}\}$ is the variance in the imaginary ionization time of the direct (scattered) electron trajectory, respectively (taken as the full dynamic range of this parameter given by the SFA). We find that the error caused by this mechanism is less than 0.1 rad. The full error of the reconstruction, shown in Fig. 4b of the main text, integrates these effects.

Our experimental results show a good agreement, within the experimental error bar, between the experimentally reconstructed difference in the ionization times and the theoretical prediction by the SPA. Importantly, corrections to the SPA will shift the locations of destructive and constructive interference in the electron hologram. However, in our analysis we extract these locations from the experimental hologram directly, and therefore compensate for such corrections.

Supplementary Note 8: Experimental set up

Electron holograms in our experiment were generated using 30fs pulses, centered around 788nm at 1kHz repetition rate, reaching a peak intensity of $1.3 \times 10^{14}\,\mathrm{W/cm^2}$. The perturbative second harmonic (SH) field is produced using a 200 μm Type I $\beta - \mathrm{BaB_2O_4}$ crystal. The conversion efficiency is controlled by altering the phase-matching conditions through crystal rotation, while avoiding elliptical polarization. The SH field is initially orthogonally polarized with respect

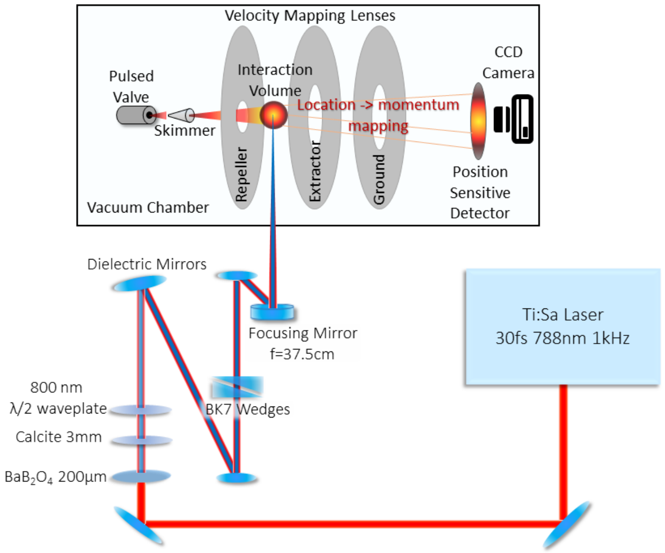

Supplementary Figure 5: The two color electron holography experimental setup. The second harmonic is produced using a 200 μm Type I $\beta - \mathrm{BaB_2O_4}$ crystal. The sub-cycle delay of the SH relative to the fundamental field is controlled using a pair of fused silica wedges.

to the fundamental field. With the two colors in orthogonal polarization, the group velocity dispersion (GVD) is compensated using calcite windows. The polarizations are then made parallel using a 800nm zero-order half-wave plate (λ/2). The sub-cycle delay of the SH relative to the fundamental field is controlled using a pair of fused silica wedges. The beams are focused into a pulsed Argon jet using a curved mirror ($\mathrm{f} = 37.5\,\mathrm{cm}$). We used a piezo valve at 100 Hz repetition rate with 1.5 bar backing pressure and an opening time of 150 μs. The ionized photoelectrons are detected using a velocity map imaging (VMI) spectrometer. In the VMI, the photoelectrons are mapped using electrostatic lenses according to their momentum onto a micro-channel plate coupled to a phosphor screen that is recorded with a CCD camera [8]. A schematic drawing of the experimental setup is shown in supplementary figure 5.

Supplementary References

[1] Keldysh, L. *et al.* Ionization in the field of a strong electromagnetic wave. *Sov. Phys. JETP* **20**, 1307–1314 (1965).

[2] Faisal, F. H. Multiple absorption of laser photons by atoms. *Journal of Physics B: Atomic and Molecular Physics* **6**, L89 (1973).

[3] Reiss, H. R. Effect of an intense electromagnetic field on a weakly bound system. *Physical Review A* **22**, 1786 (1980).

[4] Lewenstein, M., Balcou, P., Ivanov, M. Y., Lhuillier, A. & Corkum, P. B. Theory of high-harmonic generation by low-frequency laser fields. *Physical Review A* **49**, 2117 (1994).

[5] Ivanov, M. Y., Spanner, M. & Smirnova, O. Anatomy of strong field ionization. *Journal of Modern Optics* **52**, 165–184 (2005).

[6] Huismans, Y. *et al.* Time-resolved holography with photoelectrons. *Science* **331**, 61–64 (2011).

[7] Popruzhenko, S. & Bauer, D. Strong field approximation for systems with coulomb interaction. *Journal of Modern Optics* **55**, 2573–2589 (2008).

[8] Eppink, A. T. & Parker, D. H. Velocity map imaging of ions and electrons using electrostatic lenses: Application in photoelectron and photofragment ion imaging of molecular oxygen. *Review of Scientific Instruments* **68**, 3477–3484 (1997).
